# Socioeconomic inequalities in blood pressure: co-ordinated analysis of 147,775 participants from repeated birth cohort and cross-sectional datasets, 1989 to 2016

**DOI:** 10.1186/s12916-020-01800-w

**Published:** 2020-11-18

**Authors:** David Bann, Meg Fluharty, Rebecca Hardy, Shaun Scholes

**Affiliations:** 1 Centre for Longitudinal Studies, Social Research Institute, University College London, London, UK; 2 CLOSER, Social Research Institute, University College London, London, UK; 3 Department of Epidemiology and Public Health, University College London, London, UK

**Keywords:** Blood pressure, Hypertension, Socioeconomic inequality, Social determinants of health

## Abstract

**Background:**

High blood pressure (BP) is a key modifiable determinant of cardiovascular disease and a likely determinant of other adverse health outcomes. While socioeconomic inequalities in BP are well documented, it remains unclear (1) how these inequalities have changed across time, given improvements over time in the detection and treatment of high BP (hypertension); (2) whether BP inequalities are present below and above hypertension treatment thresholds; and (3) whether socioeconomic position (SEP) across life has cumulative effects on BP. We sought to address these gaps using evidence from two complementary sources: birth cohort and repeated cross-sectional datasets.

**Methods:**

We used three British birth cohort studies—born in 1946, 1958, and 1970—with BP measured at 43–46 years (in 1989, 2003, and 2016), and 21 repeated cross-sectional datasets—the Health Survey for England (HSE), with BP measured among adults aged ≥ 25 years (1994–2016). Adult education attainment was used as an indicator of SEP in both datasets; childhood father’s social class was used as an alternative indicator of (early life) SEP in cohorts. Adjusting for the expected average effects of antihypertensive medication use, we used linear regression to quantify SEP differences in mean systolic BP (SBP), and quantile regression to investigate whether inequalities differed across SBP distributions—below and above hypertension treatment thresholds.

**Results:**

In both datasets, lower educational attainment was associated with higher SBP, with similar absolute magnitudes of inequality across the studied period. Differences in SBP by education (Slope Index of Inequality) based on HSE data were 3.0 mmHg (95% CI 1.8, 4.2) in 1994 and 4.3 mmHg (2.3, 6.3) in 2016. Findings were similar for diastolic BP (DBP) and survey-defined hypertension. Inequalities were found across the SBP distribution in both datasets—below and above the hypertension threshold—yet were larger at the upper tail; in HSE, median SBP differences were 2.8 mmHg (1.7, 3.9) yet 5.6 mmHg (4.9, 6.4) at the 90th quantile. Adjustment for antihypertensive medication use had little impact on the magnitude of inequalities; in contrast, associations were largely attenuated after adjustment for body mass index. Finally, cohort data suggested that disadvantage in early and adult life had cumulative independent associations with BP: cohort-pooled differences in SBP were 5.0 mmHg (3.8, 6.1) in a score combining early life social class and own education, yet were 3.4 mmHg (2.4, 4.4) for education alone.

**Conclusion:**

Socioeconomic inequalities in BP have persisted from 1989 to 2016 in Britain/England, despite improved detection and treatment of high BP. To achieve future reductions in BP inequalities, policies addressing the wider structural determinants of high BP levels are likely required, particularly those curtailing the obesogenic environment—targeting detection and treatment alone is unlikely to be sufficient.

## Background

High blood pressure (BP) is a key modifiable determinant of cardiovascular disease, with higher risk continuing across its distribution beyond hypertension treatment thresholds [1]. Cardiovascular diseases in turn remain leading causes of mortality worldwide [2], with substantial public health and associated economic costs. Higher BP—particularly in midlife [3, 4]—is also implicated in the occurrence of dementia and related neurological outcomes [5, 6]. As such, monitoring of BP at the population level is important, as is understanding its socioeconomic inequality given its likely contribution to inequities in cardiovascular disease morbidity and mortality [7]. While there is repeated evidence that more disadvantaged socioeconomic position (SEP) is correlated with, and potentially causally linked with, higher BP [8–13], important gaps in understanding remain.

First, it is unclear how associations between SEP and BP have changed across time—and as such whether previous policies have effectively reduced such inequalities. In the UK and other high-income countries, average BP levels have declined from the 1970s onwards [14]—a change typically attributed to decreased salt intake in foods [15] and improved hypertension awareness, detection, and treatment [16]. How these and other changes such as secular increases in body mass index (BMI) (itself socially patterned) [12, 17] have impacted on socioeconomic inequalities in BP, however, remains unclear. The most recent UK analysis of trends in BP inequalities used repeated cross-sectional data from England in 1994–2008 [12] and requires updating using more recent data. It is also likely limited by (1) using an indirect area-based SEP indicator, which may be biased due to measurement error or socioeconomic and health-related migration [18, 19], and (2) not accounting for use of antihypertensive medication—an important consideration since increases over time in levels of treatment for high BP in England and worldwide [16, 20] may have narrowed or widened inequalities in BP, depending on its differential impacts.

Second, there are uncertainties regarding the life course nature of inequalities in BP—that is, when in life exposure to disadvantaged SEP impacts on adult BP outcomes. Existing studies have suggested that there are cumulative effects of disadvantaged SEP across life on higher BP (i.e. that there are independent effects of exposure to disadvantage in both child and adult life) [21], while other studies have suggested that childhood SEP is particularly important for adult BP (among men) [22]. This evidence is based on analysis conducted in separate British birth cohorts using different empirical strategies [21–24]. In contrast, a co-ordinated analytic approach across different cohorts can increase statistical power, improve result generalisability, and enable testing of whether such processes have changed across time.

Finally, most existing studies have documented SEP differences in average levels of BP (via linear regression) or differences in the binary outcome of hypertension (via logistic regression) [13], with no examination of whether inequalities differ across the BP distribution. As Geoffrey Rose indicated in 1985 [25], considering distributions is important to gain an understanding of the types of interventions which are likely to most effectively improve population health. If inequalities are only present at the upper tail of the BP distribution—i.e. above treatment thresholds for hypertension—then focusing efforts to improve the awareness, treatment, and control of hypertension may be expected to reduce such inequalities. However, if inequalities are present across the entire BP distribution, then such approaches are likely to be inherently limited since even SEP differences in average levels of BP below treatment thresholds may contribute significantly to inequalities in cardiovascular disease morbidity and mortality. To address this limitation, this paper utilises quantile regression to quantify the magnitude of inequalities at different points of the BP distribution [26].

Studies of BP inequalities have used different data sources separately—such as repeated cross-sectional [12] or single birth cohort studies [10, 22, 23, 27, 28]. This study is the first, to our knowledge, to conduct co-ordinated analyses across national birth cohort studies and repeated cross-sectional surveys to examine change across time in BP inequalities. A co-ordinated approach across these different data sources has multiple advantages. First, given their independent sampling designs, the robustness of the results and generalisability to the population is likely improved. Second, they have complementary strengths which aid understanding—birth cohort studies contain prospective SEP data across life, yielding insight into when in life exposure to disadvantage may affect BP outcomes in adulthood. The availability of BP measures in midlife in each birth cohort (age 43–46 years) enables sufficiently powered investigation of how inequalities have changed across time (1989, 2003, and 2016) at this key life stage [3, 4, 29]. While lacking information on early life SEP, use of nationally representative repeated cross-sectional data enables investigation of how inequalities in BP have changed across all adults and period (annually from 1994).

Consistent with a fundamental cause model of health inequality [30], we hypothesised that absolute inequalities in BP have persisted across the studied period (1989 to 2016)—despite improvements in the detection and treatment of high BP across all social groups—since there are multiple likely mediating pathways between SEP and high BP, some of which are likely to have stubbornly persisted over the last two decades. These include persisting inequalities in BMI [12, 17], which may mediate (i.e. partially or entirely account for [31]) inequalities in BP [7, 10]. We also hypothesised that such inequalities would be largest at the upper tail of the BP distributions, as previously found in the UK and elsewhere for BMI [26]. Finally, we hypothesised that exposure to disadvantage in both early and adult life would have persisting additive cumulative associations with higher BP.

## Methods

We used two data sources: (1) three birth cohort studies and (2) 21 repeated cross-sectional studies. For each, participants gave verbal and/or written consent to be interviewed, to be visited by a nurse, and to have BP measurements taken during a home visit. Research ethics approval was obtained from relevant committees.

### Birth cohorts

Each cohort was designed to be nationally representative when initiated in 1946 (MRC National Survey of Health and Development [32, 33]—1946c), 1958 (National Child Development Study [34]—1958c), and 1970 (British Cohort Study [35]—1970c). The history, design, and characteristics of these studies have been previously described in detail in papers [32–36] and books [37, 38]; studies have also examined the characteristics of those lost due to attrition [39–42]. The analytical sample sizes were those with valid adult BP data, measured in each study from age 43–46 years—total *N* = 18,657: 1946c = 3186, 1958c = 8610, and 1970c = 6861. A flowchart summarising derivation of the analytical sample sizes is shown in Additional file 1: Fig. S1. All analyses using the 1946c were weighted to account for the stratified sampling design [33]. To reduce the potential impact of missing data on statistical power and the potential for selection bias in longitudinal analyses, missing SEP data for those with valid BP data were replaced using multiple imputation using chained equations (*n* = 10 datasets; analyses using imputed data are presented) [43].

#### Measurement of blood pressure

Trained nurses measured resting BP using standardised protocols via sphygmomanometers, following a rest time of 5 min. Specifically, in 1946c, BP was measured twice using the Hawksley random zero sphygmomanometer in the right arm; in 1958c, three times using an Omron 705CP BP monitor in the left arm; and in 1970c, three times using an Omron HEM 907 BP monitor in the right arm. To account for differences in device, as Omron devices provide higher estimates than sphygmomanometers, the 1946c readings were converted to automated Omron readings via a previously derived formulae [44]. Pregnant women were excluded from BP measurement in the 1958c and 1970c. To aid between-cohort comparability, the second BP measure was used, or the first if the second was missing. Use of BP-lowering medication was derived using a single item on self-reported use of hypertension medication in 1946c and coded from medication data in 1958c and 1970c using British National Formulary codes [45].

### Repeated cross-sectional studies

The Health Survey for England (HSE) is a cross-sectional, general population survey of individuals living in private households, with a new sample each year randomly selected by address [46]. Data collection occurs throughout the year, via a health interview followed by a nurse visit. Twenty-one sweeps of the HSE were used for the present analysis spanning 1994 to 2016—BP data were available annually, except 1999 and 2004. The percentage of eligible households taking part in the HSE ranged from 79% in 1996 to 59% in 2016 (Additional file 1: Fig. S1). The analytical sample included *n* = 129,118 non-pregnant adults aged 25 years and over with valid BP, medication, and education data; 25 was chosen as the lower age limit to avoid bias in the classification of highest educational attainment (see below). This represented 65% of all adult participants. Starting from 2003, weights have been created to minimise bias from non-response; the relevant weights for analysing BP data were therefore used from 2003 onwards.

#### Measurement of blood pressure

BP was measured using standardised protocols with the use of Dinamap 8100 monitors before 2003, and Omron HEM 907 from 2003 onwards [20]. We converted Dinamap readings into Omron readings using a regression equation based on a calibration study [20]. Across all survey years, three BP readings were taken from each participant in a seated position at 1-min intervals with use of an appropriately sized cuff on the right arm if possible after a 5-min rest. Participants who had exercised, eaten, drunk alcohol, or smoked in the 30 min before measurements were excluded from analyses. The mean of the second and third readings was used across all survey years. Details of which, if any, classes of antihypertensive medications were being taken were recorded by the nurse.

### Socioeconomic position ascertainment

Given evidence in a recent meta-analysis for the particular relevance of education for social gradients in BP [13], and evidence for its potential causal role [7, 9], it was the main SEP indicator in this study. Education for HSE participants was measured as the highest qualification attained (at the time of interview). For the birth cohort studies, own highest education attainment was ascertained at ages 26 (1946c), 34 (1958c), and 33 (1970c). For both data sources, highest educational attainment was categorised into four groups as follows: university degree or higher, A levels/diploma, O levels/GCSEs/vocational equivalent, or none [47]. These equate to other ordered education attainment measures used in other countries—equivalent to college, advanced high school, basic high school, and no high school, respectively.

In addition to education, a comparable indicator of early life (childhood) SEP was used in analyses of birth cohort data. This was measured by father’s social class at age 4 in 1946c (occupation at birth was not used to avoid WWII-related misclassification), and at birth in 1958c and 1970c. Data at 10/11 years was used if missing at the earlier age. The Registrar General’s Social Class Scale was used with categories as follows—I (professional), II (managerial and technical), IIIN (skilled non-manual), IIIM (skilled manual), IV (partly skilled), and V (unskilled) occupations. To examine cumulative associations (see below), the ridit scores for social class at childhood and own education were combined into a single score and re-scaled.

### Analytical strategy

The analytical sample size was 147,775—those with BP outcomes at age 43–46 years (cohorts) or 25 years and over (HSE). The primary outcome for our study chosen a priori was the continuous level of systolic BP (SBP). In addition, analyses were repeated for two secondary outcomes: diastolic BP (DBP) and the binary outcome of survey-defined hypertension (SBP ≥ 140 mmHg or DBP ≥ 90 mmHg [48] or reported use of BP-lowering medication).

Use of antihypertensive medications potentially complicates the analysis of BP levels and changes in BP over time. Participants who reported use of BP-lowering treatment were likely to have observed BP values that were lower than their “underlying” BP values. Based on estimated average treatment effects, we adjusted for the expected average effects of antihypertensive medication use on BP by adding a constant of 10 mmHg (SBP) and 5 mmHg (DBP) to the observed BP values among those on treatment to approximate “underlying” BP (i.e. the BP individuals would have if they were not on treatment) [49]; this (constant addition) method has been found to reduce bias in the estimated effect of key determinants on continuous levels of BP due to the effects of antihypertensive medication use, in contrast to adjusting for treatment as a covariate in a regression model [50, 51]. Observed BP values were used with data on use of BP-lowering medication to determine hypertension status.

#### Trends across time in socioeconomic inequalities in BP

The birth cohort and HSE datasets were analysed separately. Data from each source was analysed overall (i.e. pooled across cohorts/survey years) as well as separately by birth cohort/survey year. To provide single quantifications of inequalities in a metric recommended for use in national health inequality statistics [52], the SEP indicators described above were converted to ridit scores ranging from 0 (most advantaged) to 1 (least advantaged), with each SEP group assigned a score (fractional rank) based on the midpoint of its range in the cumulative distribution in the dataset. Use of ridit scores enables comparisons across birth cohorts and survey years while accounting for differences in the proportion of participants in each SEP category. By entering the ridit scores into the model, the SEP coefficient in linear regression—the Slope Index of Inequality (SII)—is interpreted as the estimated absolute (mean) difference (absolute inequality) in BP between the lowest and highest SEP. For survey-defined hypertension, this forms a linear probability model [53]—the absolute difference in the probability of hypertension between the lowest and highest SEP. Given the large age range in the HSE datasets, adjustment was made for age in all models (entered as a categorical variable via 10-year age bands).

No adjustment was made in the birth cohort datasets as the age differences were small and were co-linear with birth year. Our regression models were also gender-adjusted, since gender differences in SEP and BP associations were not anticipated a priori. In the birth cohorts, childhood father’s social class was used as an additional indicator of SEP, and additional adjustment for other SEP indicators was made to examine the life course nature of inequalities in BP (see below). Investigation of change across time in the SEP and BP associations was performed by comparing the magnitude (and 95% confidence intervals) of the birth cohort and survey-year specific estimates.

#### The life course nature of inequalities in BP: birth cohort analyses

To examine whether SEP across life had cumulative associations with outcomes, analyses in birth cohorts were conducted before and after mutual adjustment for childhood SEP (father’s social class), own education, and own social class at 43/46 years; mutual independence of association after adjustment provides evidence for a cumulative association. In further analyses, we used the combined score for father’s social class and own education in the regression models: a larger effect size for the combined score versus either in isolation also suggests a cumulative association.

#### Inequalities across the outcome distribution

To examine if the magnitude of inequalities differed across the BP distributions, conditional quantile regression was used [54]. Quantile regression facilitates estimation of inequalities at a given quantile of the distribution. Using the same ridit scores as described above, estimates of the SII were obtained and plotted at the 5th, 10th, 25th, 50th (median), 75th, 90th, and 95th quantiles. To facilitate interpretation, we show the quantiles corresponding to the 140/90 mmHg BP thresholds for initiating BP-lowering treatment [55].

#### The influence of antihypertensive medication

To examine how the adjustment for use of antihypertensive medication affected the direction and/or magnitude of inequalities in BP, associations between education and BP-lowering medication use were analysed using linear probability models, before and after adjustment for SBP (to indicate treatment need). Main analyses were repeated without adjustment for use of BP-lowering medication in order to estimate inequalities in observed rather than “underlying” BP.

#### The influence of body mass index

To examine the extent to which BMI contributes to BP inequalities (e.g. by attenuating the SEP-BP associations through likely mediation), associations between SEP and SBP outcomes were estimated before and after adjustment for BMI. BMI (weight in kilogrammes divided by height in metres squared) was ascertained at the same age as BP measures—it was calculated using height and weight measures obtained by trained interviewers using standardised protocols, as described elsewhere [12, 17].

#### Additional and sensitivity analyses

To examine the robustness of associations of early life SEP with BP—to a different parental respondent and different dimension of disadvantage—maternal educational status was used instead of father’s social class. This was measured via a binary indicator of whether the participant's mother had left formal education at the mandatory leaving age (14 years old from 1918, 15 from 1944, and 16 from 1972). To examine whether differences in missing outcome data may have biased cross birth-cohort study estimates, analyses were repeated using multiple imputation to additionally impute BP outcomes in the eligible target population (i.e. those alive and who had not emigrated). Gender-specific analyses were conducted to examine if any change over time in BP inequalities differed by gender. Likewise, age-specific analyses (25–54, 55+) using the HSE datasets were conducted to examine if age modified the magnitude of inequalities.

## Results

Comparing 2016 with previous years, both cohort and HSE datasets indicated increased higher educational attainment, more prevalent use of BP-lowering medication, and lower mean SBP and DBP (Table 1; differences in SBP in cohort data were more modest than those observed in HSE). Cohort data indicated that the percentage of participants with fathers of manual social class decreased across time (Table 1). The prevalence of outcome missingness in the cohorts, attributable to survey loss to follow-up, migration, and premature mortality from birth to 43–46 years, was higher in each subsequent cohort (40.6% in 1946c, 50.1% in 1958c, and 57.7% in 1970c).

**Table 1:** Participant characteristics: data from 3 British birth cohort studies and 21 repeated cross-sectional English studies

	Birth cohort study in midlife (43–46 years), year of outcome measurement (birth year)			Repeated cross-sectional study (≥ 25 years), year of outcome measurement		
	1989 (1946)	2003 (1958)	2016 (1970)	1994	2003	2016
**Outcomes**						
Sample size	3186	8610	6861	11,427	7910	3981
Systolic blood pressure, mean (SD) mmHg*	125.8 (15.5)	126.4 (17.0)	124.1 (15.5)	133.6 (18.3)	129.9 (19.1)	125.9 (17.2)
Diastolic blood pressure, mean (SD) mmHg*	81.6 (10.4)	78.8 (11.2)	77.1 (11.2)	75.1 (8.9)	74.9 (11.5)	73.5 (11.3)
Hypertension, %**	23.8%	28.4%	24.5%	35.5%	33.8%	31.6%
Blood pressure-lowering medication, %	3.8%	6.1%	8.1%	13.5%	14.3%	17.9%
**Socioeconomic data**						
Social class at birth–4years, % manual	74.4%	72.7%	70.8%	–	–	–
Own education attainment, % degree	6.6%	8.4%	24.1%	11.1%	19.3%	32.0%

With valid BP, medication, and SEP data

*Observed BP values (i.e. not adjusted for use of antihypertensive medication); HSE sample size is unweighted, and estimates are weighted

**Hypertension: SBP/DBP ≥ 140/90 mmHg and/or on BP-lowering medication

### Trends across time in socioeconomic inequalities in BP

**Fig. 1:**
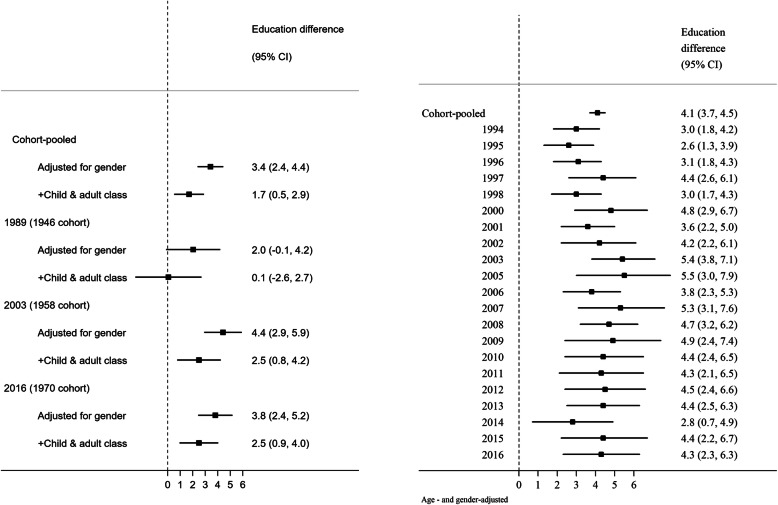
Education attainment and mean difference in systolic blood pressure (mmHg) in midlife (43–46 years, from birth cohort data, left panel) and across adulthood (≥ 25 years, from repeated cross-sectional data, far right panel). Estimates are the Slope Index of Inequality (absolute difference in mean SBP levels between the lowest and highest socioeconomic position). An SII of zero (vertical line) indicates equity in BP levels. Underlying SBP levels obtained by adding a constant of 10 mmHg to those using antihypertensive medication. Estimates adjusted for child and adult social class indicate potential cumulative associations over the life course

**Fig. 2:**
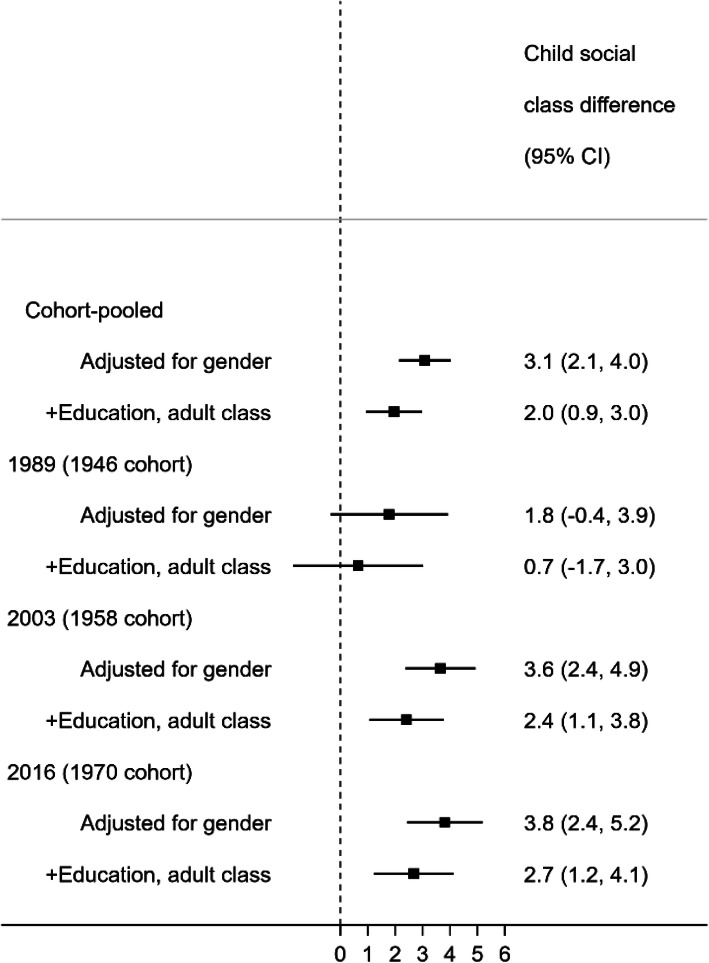
Socioeconomic position in early life and mean difference in systolic blood pressure (mmHg) in midlife (43–46 years, from birth cohort data). Estimates are the Slope Index of Inequality (absolute difference in mean SBP levels between the lowest and highest socioeconomic position). An SII of zero (vertical line) indicates equity in BP levels. Underlying SBP levels obtained by adding a constant of 10 mmHg to those using antihypertensive medication. Estimates adjusted for own education and adult social class indicate potential cumulative associations over the life course

In both cohort and HSE datasets, lower education was associated with higher SBP (Fig. 1). In HSE, the education-related (SII) difference in SBP based on data pooled across the 21 surveys was 4.1 mmHg (95% CI 3.7, 4.5). The magnitude of the social gradient in BP outcomes changed little across time; estimates of absolute inequality in later years were typically larger than earlier years but 95% CIs overlapped (with larger margin of error in later years due to smaller sample sizes). For example, in HSE, the SII in SBP was 3.0 mmHg (95% CI 1.8, 4.2) in 1994 and 4.3 mmHg (2.3, 6.3) in 2016. Estimates for 1946c were somewhat smaller than 1958c and 1970c, yet 95% CIs overlapped (Fig. 1). Using an alternative indicator of SEP, disadvantaged early life SEP (father’s social class) was also associated with higher SBP in each cohort, with similar patterns of association as with education (Fig. 2). These findings of persistent inequalities across time in SBP were similar for DBP and for survey-defined hypertension (Additional file 1: Fig. S2).

### The life course nature of inequalities in BP

Lower own educational attainment and more disadvantaged childhood social class were associated with higher SBP, before and after adjustment for each SEP indicator and own adult social class (Figs. 1 and 2, respectively). These adjusted associations were weaker in 1946c than subsequent cohorts, yet 95% CIs overlapped, and as expected, were most precisely estimated in cohort-pooled models (Figs. 1 and 2, top row). As anticipated given evidence for their independent associations, associations between the composite score (childhood social class and own education) and SBP yielded higher effect estimates than either in isolation: in a cohort-pooled model, the difference in SBP was 5.0 mmHg (3.8, 6.1) using the composite score yet 3.4 mmHg (2.4, 4.4) when considering education alone (Fig. 1), and 3.1 mmHg (2.1, 4.0) when considering childhood social class alone (Fig. 2). Findings were similar for DBP and survey-defined hypertension (Additional file 1: Fig. S2).

### Inequalities across the distribution of BP

**Fig. 3:**
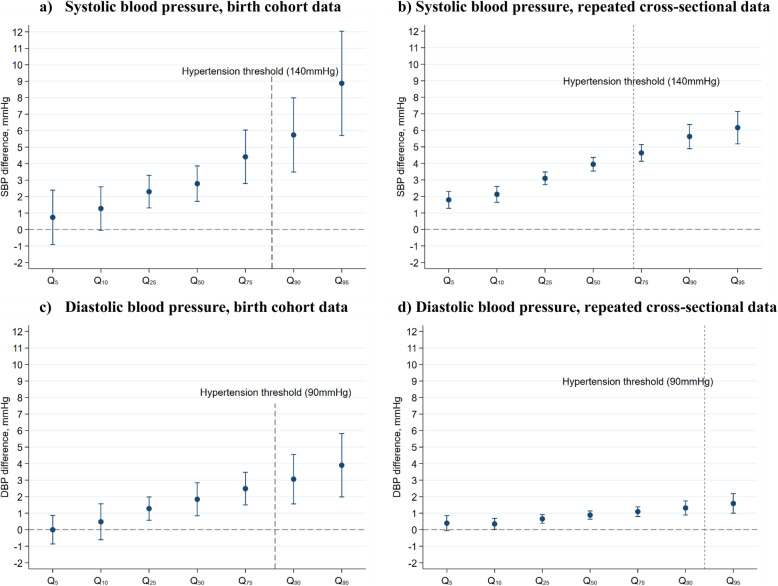
Estimated differences in systolic and diastolic blood pressure in the lowest versus highest education attainment (Slope Index of Inequality): quantile regression estimates at different quantiles of the outcome distribution (95% CI). Coefficients are interpreted analogously to linear regression: for example, Q_50_ shows the median difference in BP comparing the lowest with highest education attainment. An SII of zero (horizontal line) indicates equity in BP levels. Underlying SBP and DBP levels obtained by adding a constant of 10 and 5 mmHg to those using antihypertensive medication, respectively. The quantiles of the BP distribution corresponding to the hypertension thresholds calculated by estimating the proportion of participants with BP values below 140/90 mmHg. Q_5_ estimate for DBP in birth cohorts: the estimate and its 95% CI were not obtained since the analytical model did not converge (due to insufficient outcome variance at that quantile)

In both HSE and birth cohorts, quantile regression analyses revealed education-related differences in SBP and DBP across the BP distributions—below and above the 140/90 mmHg hypertension thresholds. However, the estimated mean differences in SBP and DBP were likely driven by the larger effect sizes at the upper tail of the distribution (Fig. 3). For example, in a cohort-pooled model, the mean difference in SBP was 1.3 mmHg (0.0, 2.6) at the 10th quantile, 2.8 mmHg (1.7, 3.9) at the median, and 5.7 mmHg (3.5, 8.0) at the 90th quantile (Fig. 3). Estimates from pooled HSE analyses were 2.1 mmHg (1.6, 2.6), 3.9 mmHg (3.5, 4.3), and 5.6 mmHg (4.9, 6.4), respectively. We found similar results albeit with lower precision when conducted in each birth cohort and HSE dataset separately; data available upon request.

### The influence of antihypertensive medication

Lower education was associated with increased likelihood of using BP-lowering medication (before and after adjustment for measured SBP)—the education-related (SII) difference in antihypertensive medication use after adjustment for SBP was 3.8 (2.4, 5.2) percentage points in a cohort-pooled model and 4.6 (3.9, 5.3) in HSE (Additional file 1: Fig. S3). Main findings were similar when not adjusting BP levels for use of BP-lowering medication, yet the magnitude of SEP differences in observed rather than “underlying” SBP—as expressed by the SII—was lower in magnitude in each data source (Additional file 1: Fig. S4).

### The influence of body mass index

In both HSE and birth cohorts, SEP differences in BP outcomes were largely attenuated after adjustment for BMI in the regression models. For example, in a cohort-pooled model, education-related (SII) differences in SBP were 3.4 mmHg (95% CI 2.4, 4.4) before and 1.8 mmHg (95% CI 0.8, 2.9) after adjustment for BMI—an attenuation in the SII estimate of almost 50% (Additional file 1: Fig. S5). The corresponding estimates from pooled HSE analyses were 4.5 mmHg (2.4, 6.7) and 2.6 mmHg (0.6, 4.7), respectively (Additional file 1: Fig. S5).

### Additional and sensitivity analyses

Findings were similar when maternal education was used as an indicator of childhood SEP instead of father’s social class—in a birth cohort-pooled model, the SEP difference in SBP according to maternal education alone was 3.9 mmHg (2.8, 5.0), and 5.6 mmHg (4.3, 6.9) in a score combining maternal and own education. Findings were also similar when imputing cohort SBP data (data available upon request), and when conducted separately in men and women (Additional file 1: Fig. S6; in HSE, the magnitude of SEP differences in SBP was larger in females, but the persistence of associations across survey years was found in both genders). Exploratory analyses in the pooled and year-specific HSE datasets by age group (25–54, 55+) suggested that age did not substantially modify the persistence of inequalities in SBP (Additional file 1: Fig. S7).

## Discussion

### Main findings

Using data from three birth cohorts and 21 repeated cross-sectional surveys—with data spanning 1989 to 2016—we found persisting socioeconomic inequalities on the absolute scale in adult SBP, DBP, and survey-defined hypertension. Our use of quantile regression found that average differences in SBP and DBP were driven by SEP differences in the upper tail of the BP distributions—i.e. higher inequalities among those at highest risk of cardiovascular disease. SEP differences in BP were still present, albeit slightly weaker, when we did not adjust for antihypertensive medication use (i.e. analysis of observed rather than “underlying” BP). In contrast, associations were markedly attenuated (~ 50%) upon adjustment for BMI. Finally, our analysis of birth cohort data suggests evidence for persisting cumulative associations of more disadvantaged SEP across life on these outcomes.

### Comparison with previous findings

Our findings of persistent inequalities in BP extend prior analyses of area-based SEP differences in high BP using HSE data (1994–2008) [12], and studies which identified mean differences in BP using 1946c or 1958c [21–24]. Our effect estimates for 1946c were smaller than those previously reported, a finding which we found attributable to our use of weights to account for the stratified sampling design in this cohort [33]. While a previous study using 1946c concluded that child SEP may be particularly important for adult SBP (among men) [22], it used social class only (not education) and a different statistical approach. Our co-ordinated analyses found consistent evidence—across 1958c and 1970c and among both genders—for cumulative associations of early life social class and adult education on BP outcomes.

Our findings also add to evidence from other countries—many of which, as in the UK, have also experienced declining average BP levels and increases in higher education attainment. Such evidence is typically derived from repeated cross-sectional studies investigating inequalities in the binary outcome of hypertension (e.g. in the USA [56], South Korea [57], Norway [58]) and/or average BP levels (e.g. in Sweden [59]) according to indicators of adult socioeconomic circumstances. Both the magnitude of SEP differences in BP and their change across time are likely to be context-specific and may therefore differ by country. Our findings are arguably most generalisable to other high-income countries which have universal or near-universal healthcare systems but show persistent inequalities in the determinants of BP such as physical inactivity, poor diet, psychosocial factors, and BMI [8], which are likely key drivers of BP inequalities. The greatest burden of hypertension however is arguably in low-income and middle-income countries. It is feasible that the magnitude of SEP inequalities in BP is larger in countries without universal healthcare coverage and/or where lower SEP groups face significant barriers to accessing healthcare and preventative medicine [60].

A recent meta-analysis suggested that SEP and high BP associations were mainly evident for associations with educational attainment [13]; our findings suggest that sizable inequalities exist due to early life socioeconomic circumstances, independently of education and adult social class. The magnitude of inequalities in BP outcomes may therefore be substantially underestimated when solely examined using education or other adult SEP indicators; this may have implications for future studies which seek to either monitor or reduce inequalities in BP outcomes.

### Explanation of findings

Effective policies to reduce inequalities improve health across all groups but with higher levels of improvement among the disadvantaged groups. The apparent persistence of inequalities observed in our study suggests that the population-level declines in average levels of BP from 1989 to 2016 (e.g. 7.7 mmHg and 1.7 mmHg for SBP and DBP respectively based on HSE data; Table 1) did not substantially differ by SEP. Multiple policies have been enacted across this period which could have feasibly affected such inequalities. These include the attempts to standardise and improve the management of chronic diseases such as BP screening in 2009 (the NHS Cardiovascular Health Check programme in England for those aged 40–74 years), and the introduction of financial incentives for UK general practitioners to regularly monitor BP in hypertensive patients in 2004. Analysis of pooled HSE data (2011 to 2016) reassuringly found equity in the key indicators of hypertension management (diagnosis, treatment, and control) [61]; nevertheless, such equity co-existed with income-based inequalities in hypertension prevalence.

Regardless of the impact of each specific policy [62, 63], there are multiple reasons why increases in detection and treatment of high BP are unlikely sufficient to reduce SEP inequalities in BP: first, treatment is not applicable to untreated adults below the BP thresholds, in which SEP differences in BP exist (as presented in this study; Fig. 3); second, among adults with hypertension, those of more disadvantaged SEP are likely to have higher BP levels, such that treatment—while potentially achieving therapeutic targets—may not fully eliminate absolute differences in BP levels across SEP groups; and third, across the studied period, inequalities in other factors that may mediate SEP and BP associations, such as higher BMI [12, 17] and diabetes [64], have likely stubbornly persisted over the last two decades, and these are expected to contribute to inequalities in BP. Consistent with this reasoning, we found that SEP differences in BP were still present, albeit slightly weaker, when we did not adjust for the expected average effects of antihypertensive medication use (i.e. analysis of observed rather than “underlying” BP). In contrast, but consistent with our reasoning, the direct associations between SEP and BP were markedly attenuated (~ 50%) upon adjustment for BMI.

Additional pathways which may persistently link SEP and BP include psychosocial or physiological processes (e.g. chronic inflammation and stress) [65], as well as other behaviours undertaken in adulthood such as smoking [66] and alcohol use [67]. Both early life and adult SEP appear to have cumulative independent associations with determinants (e.g. BMI [17]) which likely results in cumulative associations with BP in midlife. Future reductions in BP inequalities are likely to be achieved by addressing these wider determinants beyond detection and treatment which influence change in the upper tail of the BP distribution without having a major impact on average levels; these in turn are likely to require structural changes to address, such as those incentivising lower salt and calorie contents of foods (i.e. those which effectively curtail the obesogenic environment) [68].

While there is evidence (from multiple study designs) that the links between lower SEP and higher BP are causal in nature [9], bivariate trends may reflect changes in causal and/or non-causal processes. Indeed, if non-causal pathways have strengthened across time (e.g. due to increasing confounding by factors such as preceding ill health or cognition [69, 70]), they may have counteracted weakening causal effects across time, thus resulting in persisting inequalities over the study period. However, to the authors’ knowledge, there is little empirical evidence supporting the strengthening of non-causal pathways across the studied period—for instance, SEP differences in childhood cognition (a possible confounder) appear to have been similar in 1958c and 1970c [71].

We found that absolute inequalities in SBP were largely attenuated after adjustment for BMI in our models. However, as BP and BMI were measured contemporaneously, our findings do not provide strong evidence for the temporal direction of such mediation. While similar findings have been found in longitudinal analyses which adjust for BMI [10], formal mediation analyses may better account for the temporal nature of such pathways, as well as additionally account for possible common causes of associations between BMI and SEP (i.e. mediator-outcome confounders) [72].

### Strengths and limitations

Strengths of this study include the use of multiple comparable nationally representative datasets with complementary advantages. Our co-ordinated analyses of these enabled the investigation of long-run trends in SEP inequalities in BP, the incorporation of data on BP-lowering medication, and the use of multiple indicators of SEP collected in both early and adult life. The use of a single analytical framework across the 1946c and 1958c, and additionally including the 1970c, improved the available statistical power and generalisability of our investigation of the life course nature of BP inequalities. Finally, our use of quantile regression enabled us to move beyond a focus on average levels or binary outcomes to identify that such inequalities were driven by differences at the upper tail of the BP distributions. While we focused on absolute differences in BP inequality, it should be acknowledged that interpretation of trends may differ if analysed in relative terms; specifically, since average BP levels lowered across time (Table 1), and absolute differences were similar (Fig. 1), relative differences in BP outcomes by SEP are likely to have widened in the studied period.

Our study has several limitations. While we aimed to optimise the comparability of exposure and outcome data, and minimise the potential impact of missing data (via multiple imputation or weighting), we were unable to fully exclude the potential that findings, and thus inferences regarding change in the SEP and BP association across time, and its attenuation after adjustment for BMI, are biased due to unobserved predictors of missing data or unobserved methodological differences between the studies. A further limitation is that the study samples were largely White; we therefore lacked power to investigate whether inequalities and their change across time are modified by ethnicity. While multiple indicators of SEP were utilised in our birth cohort analyses, we were unable to investigate unmeasured aspects of socioeconomic circumstances which may be particularly important—such as income-related food insecurity or wealth. We accounted for the expected average effects of antihypertensive medication use on BP levels by adding a constant BP value to those on treatment; while such an adjustment has been found to reduce bias in population-level estimates of exposure-BP associations [50, 51], it is inevitably an imperfect approximation of the unobserved effects of treatment on BP levels, which may differ by regimen (e.g. factors not observed in our study such as dose and adherence). It could also potentially vary by SEP (e.g. due to socioeconomic differences in factors which modify the effect of treatment). Finally, we were unable to account for difference in adherence to prescribed antihypertensive medication. SEP differences in adherence (e.g. through health literacy and medical knowledge) could potentially exacerbate inequalities in BP and other CVD outcomes [73], though empirical research investigating such links appears to be uncertain—as evidenced by the high heterogeneity between the included studies in a recent meta-analysis [74].

## Conclusions

From 1989 to 2016 in Britain/England, socioeconomic inequalities in BP appear to have persisted in absolute terms, with additive associations of early life social class and own education. Indeed, studies examining inequalities in BP solely using education as a marker for SEP are likely to underestimate lifetime inequalities in BP outcomes. While our evidence is descriptive in nature, our findings suggest that prior strategies of reducing socioeconomic inequalities in these outcomes through wider use of BP-lowering treatments have been insufficiently effective. Alternative strategies, targeting the wider structural determinants of high BP are likely required, such as those which target the obesogenic environment.

## Supplementary information

**Additional file 1: Fig. S1.** Flow diagram showing derivation of the analytical sample size: top panel (birth cohort studies), below panel (overleaf; repeated cross-sectional studies). **Fig. S2.** Education-related difference in mean diastolic blood pressure (left panels, mmHg) and hypertension prevalence (right panels, %) in midlife (43-46 years, from birth cohort data) and across adulthood (≥25 years, from repeated cross-sectional data). **Fig. S3.** Education-related differences in blood pressure lowering medication use (%) in midlife (43-46 years, from birth cohort data, left panel) and across adulthood (≥25 years, from repeated cross-sectional data, right panel). **Fig. S4.** Socioeconomic position across life and mean difference in systolic blood pressure (mmHg) in midlife (43-46 years, from birth cohort data, left panels) and across adulthood (≥25 years, from repeated cross-sectional data, right panel); without adjusting for blood pressure-lowering treatment (i.e. observed rather than underlying BP). **Fig. S5.** Socioeconomic position and mean difference in systolic blood pressure (mmHg) in midlife (43-46 years, from birth cohort data, left panel) and across adulthood (≥25 years, from repeated cross-sectional data, far right panel)—before and after adjustment for body mass index (BMI). **Fig. S6.** Early life socioeconomic position and mean difference in systolic blood pressure (mmHg) in midlife (43-46 years, from birth cohort data, left panels) and across adulthood (≥25 years, from repeated cross-sectional data, right panel); analyses stratified by gender. **Fig. S7.** Education-related difference in mean systolic blood pressure (mmHg) in those aged 25-54 years (left panel) or 55 years and older (right panel); data from repeated cross-sectional data.

## Data Availability

1946c data are available from https://www.nshd.mrc.ac.uk/data/data-sharing/; 1958c, 1970c, and HSE data are available from the UK Data Archive: https://www.data-archive.ac.uk.
