# Supplementary material for: Socioeconomic inequalities in blood pressure: co-ordinated analysis of 147,775 participants from repeated birth cohort and cross-sectional datasets, 1989 to 2016
Source: BMC Med. 2020 Nov 18;18:338. doi: 10.1186/s12916-020-01800-w (PMC7672962; doi:10.1186/s12916-020-01800-w)
Supplement: Supplementary file 1 — Additional file 1: Fig. S1. Flow diagram showing derivation of the analytical sample size: top panel (birth cohort studies), below panel (overleaf; repeated cross-sectional studies). Fig. S2. Education-related difference in mean diastolic blood pressure (left panels, mmHg) and hypertension prevalence (right panels, %) in midlife (43-46 years, from birth cohort data) and across adulthood (≥25 years, from repeated cross-sectional data). Fig. S3. Education-related differences in blood pressure lowering medication use (%) in midlife (43-46 years, from birth cohort data, left panel) and across adulthood (≥25 years, from repeated cross-sectional data, right panel). Fig. S4. Socioeconomic position across life and mean difference in systolic blood pressure (mmHg) in midlife (43-46 years, from birth cohort data, left panels) and across adulthood (≥25 years, from repeated cross-sectional data, right panel); without adjusting for blood pressure-lowering treatment (i.e. observed rather than underlying BP). Fig. S5. Socioeconomic position and mean difference in systolic blood pressure (mmHg) in midlife (43-46 years, from birth cohort data, left panel) and across adulthood (≥25 years, from repeated cross-sectional data, far right panel)—before and after adjustment for body mass index (BMI). Fig. S6. Early life socioeconomic position and mean difference in systolic blood pressure (mmHg) in midlife (43-46 years, from birth cohort data, left panels) and across adulthood (≥25 years, from repeated cross-sectional data, right panel); analyses stratified by gender. Fig. S7. Education-related difference in mean systolic blood pressure (mmHg) in those aged 25-54 years (left panel) or 55 years and older (right panel); data from repeated cross-sectional data. [file 12916_2020_1800_MOESM1_ESM.docx]

**Supplementary Information for:**

**Socioeconomic inequalities in blood pressure: co-ordinated analysis of 147,775 participants from repeated birth cohort and cross-sectional datasets, 1989 to 2016**

David Bann^1^, Meg Fluharty^1^, Rebecca Hardy^2^, Shaun Scholes^3^

^1^Centre for Longitudinal Studies, Department of Social Science, University College London, London, UK

^2^CLOSER, Department of Social Science, University College London, London, UK

^3^Department of Epidemiology and Public Health, University College London, London, UK

**Table of Contents**

[Fig. S1. 2](#_Toc47538732)

[Fig. S2 4](#_Toc47538733)

[Fig. S3 6](#_Toc47538734)

[Fig. S4 7](#_Toc47538735)

[Fig. S5 8](#_Toc47538736)

[Fig. S6 9](#_Toc47538737)

[Fig. S7 11](#_Toc47538738)


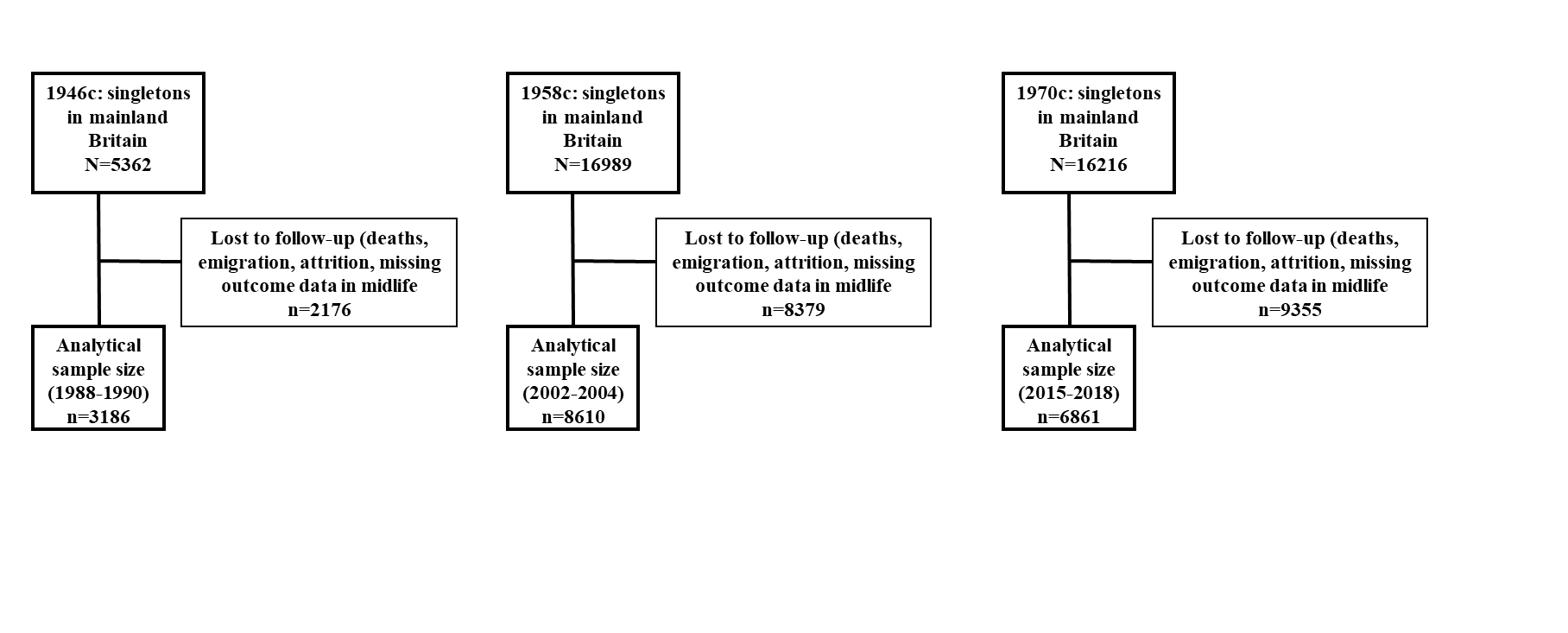


Fig. S1. Flow diagram showing derivation of the analytical sample size: top panel (birth cohort studies), below panel (overleaf; repeated cross-sectional studies).

| **Participating households (response rate)^a^:** |  | **Interviewed ^b^:** |  | **Nurse visit ^b^:** |  | **Valid blood pressure and medicine data ^b,c^:** |  | **Complete education data (analytical sample) ^b,d^:** |
| --- | --- | --- | --- | --- | --- | --- | --- | --- |
| **1994:** N=9068 (77%) | **⟶** | N=13 757 | **⟶** | N=11 999 | **⟶** | N=11 653 | **⟶** | N=11 427 |
| **1995:** N=9084 (78%) | **⟶** | N=14 040 | **⟶** | N=12 149 | **⟶** | N=11 044 | **⟶** | N= 10 427 |
| **1996:** N=9350 (79%) | **⟶** | N=14 834 | **⟶** | N=12 641 | **⟶** | N=11 532 | **⟶** | N=10 900 |
| **1997:** N=4905 (76%) | **⟶** | N=7530 | **⟶** | N=6717 | **⟶** | N=6066 | **⟶** | N=5764 |
| **1998:** N=9208 (74%) | **⟶** | N=14 027 | **⟶** | N=12 052 | **⟶** | N=10 597 | **⟶** | N=10 040 |
| **2000:** N=4788 (75%) | **⟶** | N=7134 | **⟶** | N=5989 | **⟶** | N=5070 | **⟶** | N=4847 |
| **2001:** N=9373 (74%) | **⟶** | N=13 873 | **⟶** | N=11 082 | **⟶** | N=9554 | **⟶** | N=9061 |
| **2002:** N=5112 (76%) | **⟶** | N=6436 | **⟶** | N=5217 | **⟶** | N=4287 | **⟶** | N=4051 |
| **2003:** N=8867 (73%) | **⟶** | N=13 200 | **⟶** | N=10 276 | **⟶** | N=8334 | **⟶** | N=7910 |
| **2005:** N=4546 (71%) | **⟶** | N=6728 | **⟶** | N=4897 | **⟶** | N=3907 | **⟶** | N=3822 |
| **2006:** N=8615 (68%) | **⟶** | N=12 698 | **⟶** | N=9522 | **⟶** | N=8016 | **⟶** | N=7836 |
| **2007:** N=4200 (66%) | **⟶** | N=6137 | **⟶** | N=4503 | **⟶** | N=3775 | **⟶** | N=3691 |
| **2008:** N=9191 (64%) | **⟶** | N=13 405 | **⟶** | N=9652 | **⟶** | N=8117 | **⟶** | N=7944 |
| **2009:** N=2832 (68%) | **⟶** | N=4137 | **⟶** | N=2950 | **⟶** | N=2526 | **⟶** | N=2467 |
| **2010:** N=5249 (66%) | **⟶** | N=7565 | **⟶** | N=5091 | **⟶** | N=3950 | **⟶** | N=3853 |
| **2011:** N=5338 (66%) | **⟶** | N=7755 | **⟶** | N=5225 | **⟶** | N=4042 | **⟶** | N=3979 |
| **2012:** N=5219 (64%) | **⟶** | N=7445 | **⟶** | N=4989 | **⟶** | N=4131 | **⟶** | N=4044 |
| **2013:** N=5416 (64%) | **⟶** | N=7926 | **⟶** | N=5673 | **⟶** | N=4813 | **⟶** | N=4716 |
| **2014:** N=5051 (62%) | **⟶** | N=7297 | **⟶** | N=5059 | **⟶** | N=4312 | **⟶** | N=4244 |
| **2015:** N=5111 (60%) | **⟶** | N=7325 | **⟶** | N=4977 | **⟶** | N=4191 | **⟶** | N=4114 |
| **2016:** N=5096 (59%) | **⟶** | N=7282 | **⟶** | N=4692 | **⟶** | N=4340 | **⟶** | N=3981 |
| **1994-2016** |  | **N=200,531** |  | **N=155,352** |  | **N=134,257** |  | **N=129,118** |

**Notes: ^a^**All co-operating households in HSE (i.e. at least one person interviewed): response rate calculated as co-operating households / eligible households (full details are available in HSE documentation). BP data not available in 1999 or 2004 due to the oversampling of minority ethnic groups. **^b^**Adults aged 25 years and over. ^c^Participants who had exercised, eaten, drunk alcohol or smoked in the 30 minutes before BP measurements were excluded from BP data. ^d^Education data excludes those with ‘other’ qualifications.

**…Fig. S1 continued.**


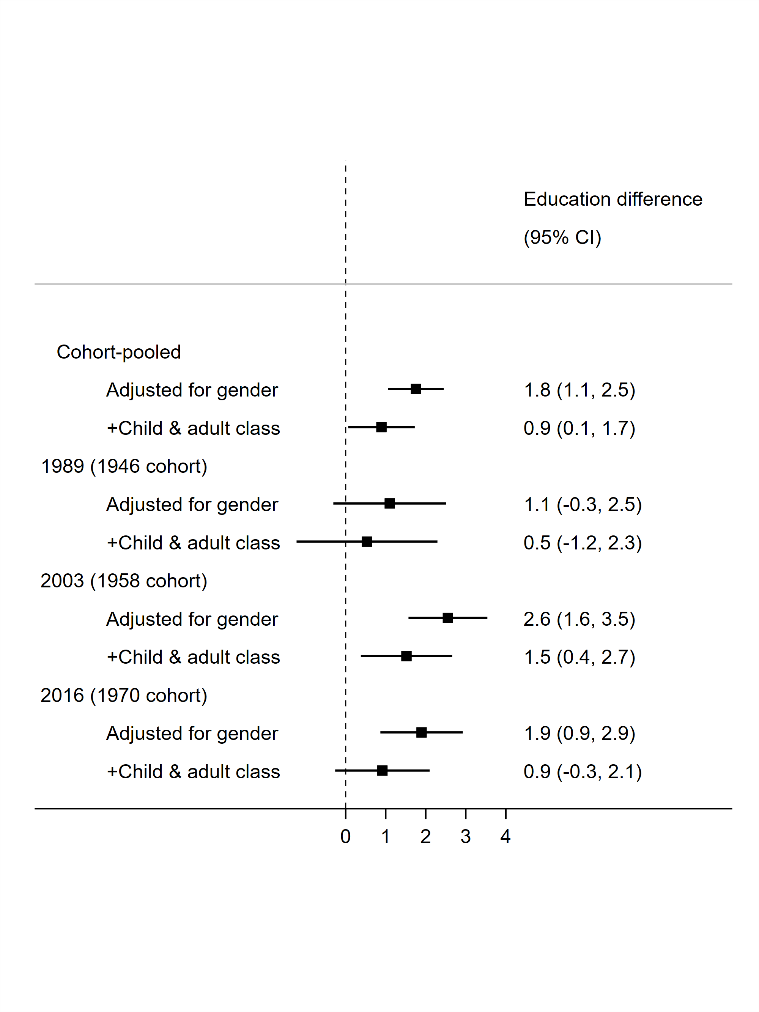

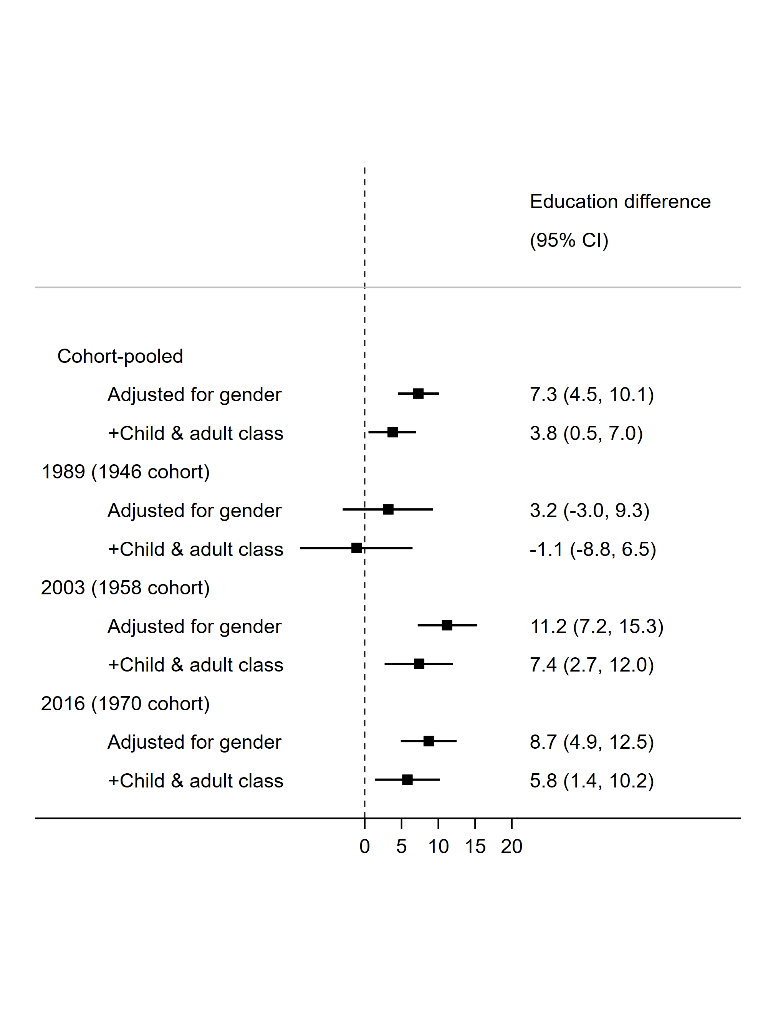


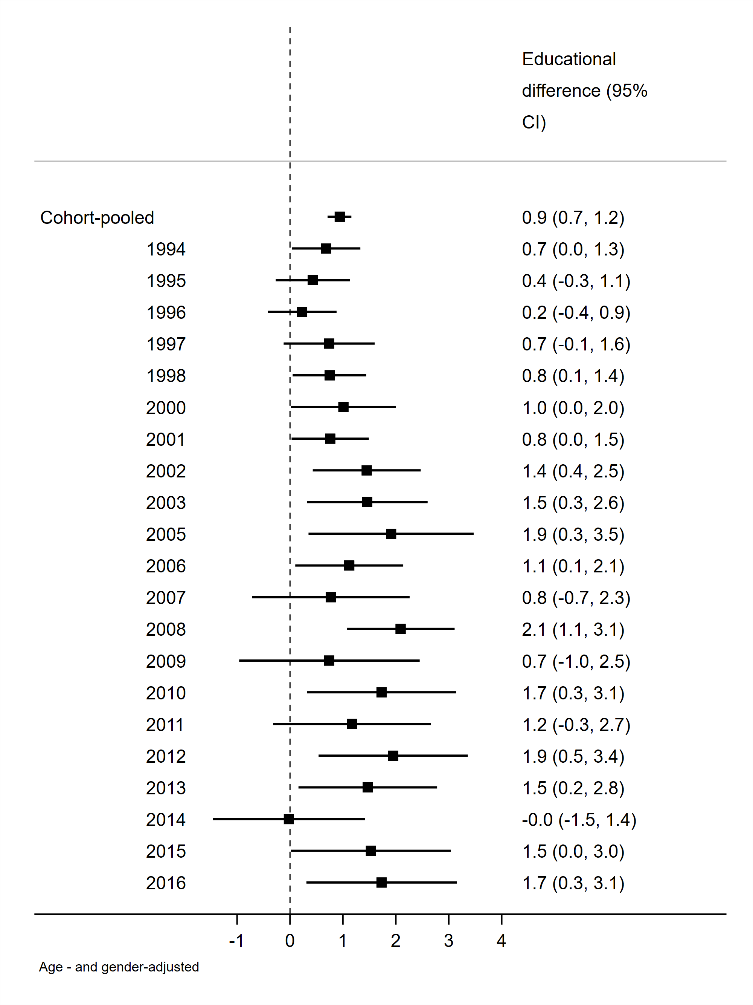

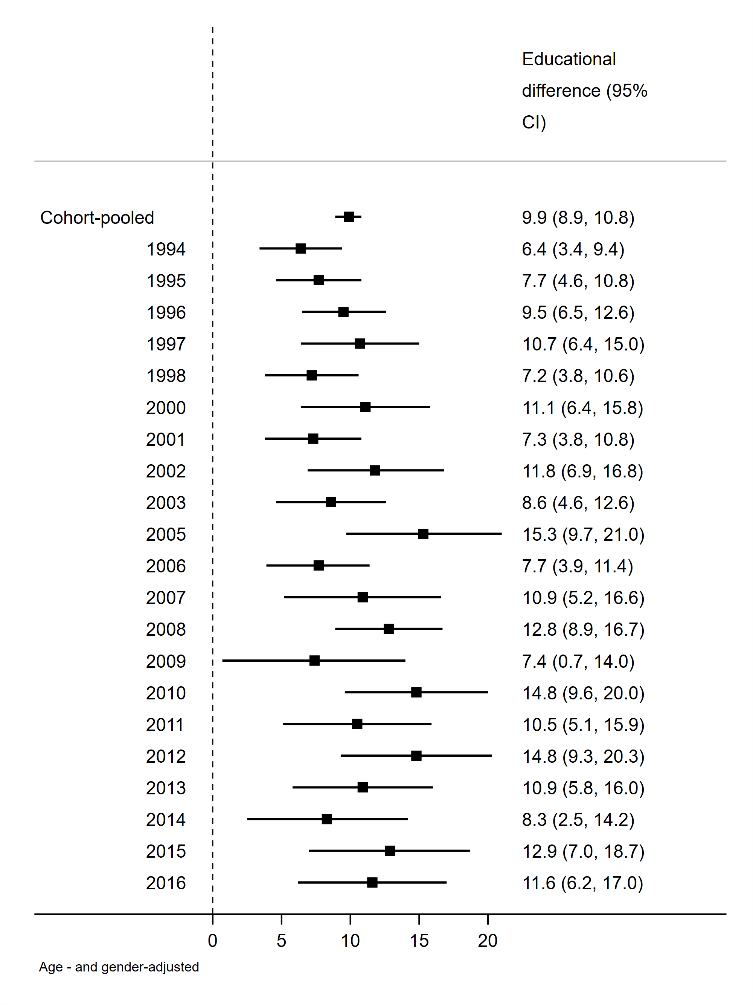


Fig. S2**. Education-related difference in mean diastolic blood pressure (left panels, mmHg) and hypertension prevalence (right panels, %) in midlife (42-46 years, from birth cohort data) and across adulthood (≥25 years, from repeated cross-sectional data).** Note: estimates are the Slope Index of Inequality (absolute difference in BP outcome between the lowest and highest educational attainment groups). An SII of zero (vertical line) indicates equity in BP outcomes. Underlying DBP levels obtained by adding a constant of 5mmHg to those using antihypertensive medication. Cohort analysis: estimates adjusted for child and adult social class indicate potential cumulative associations over the life course.


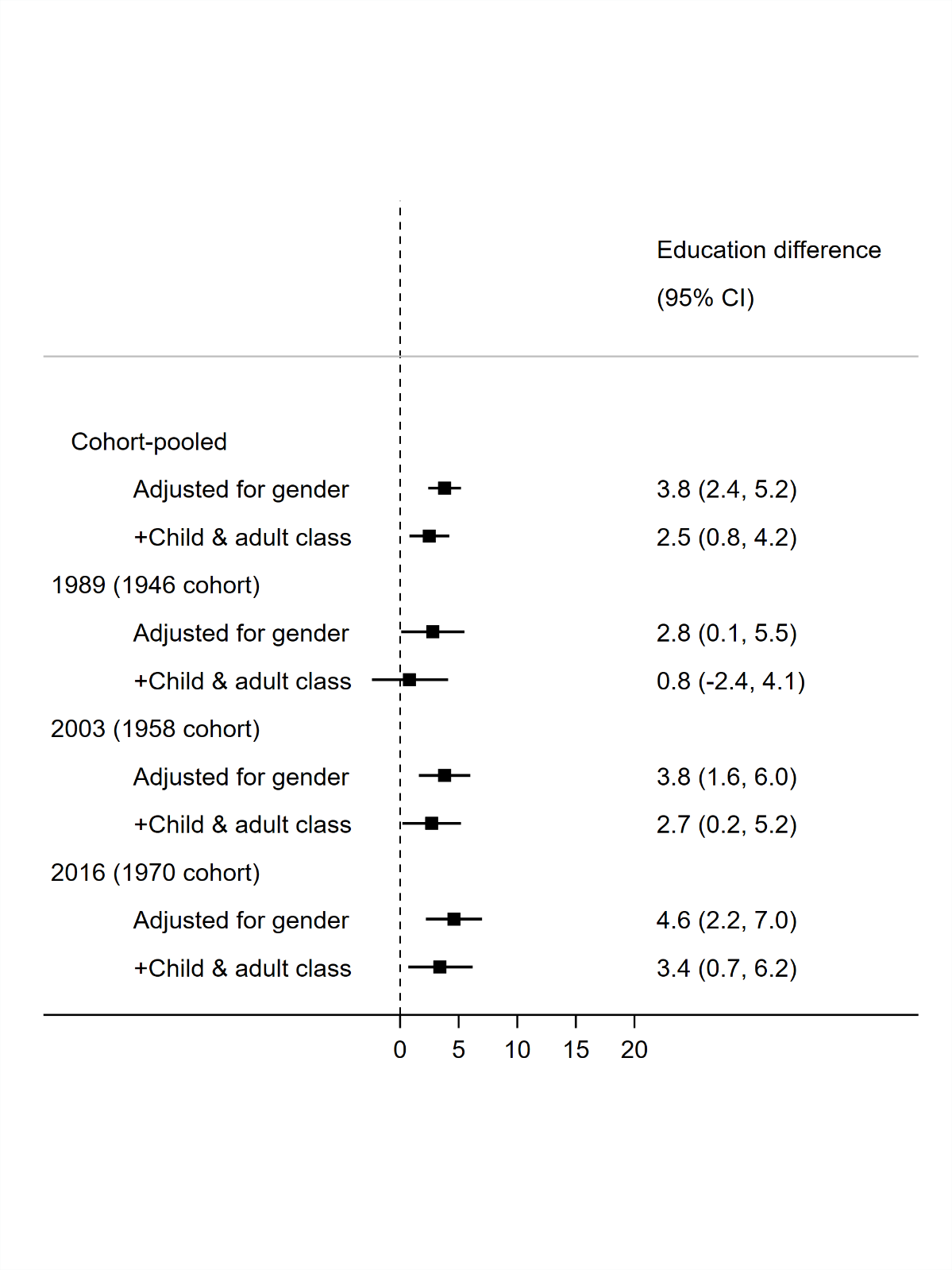

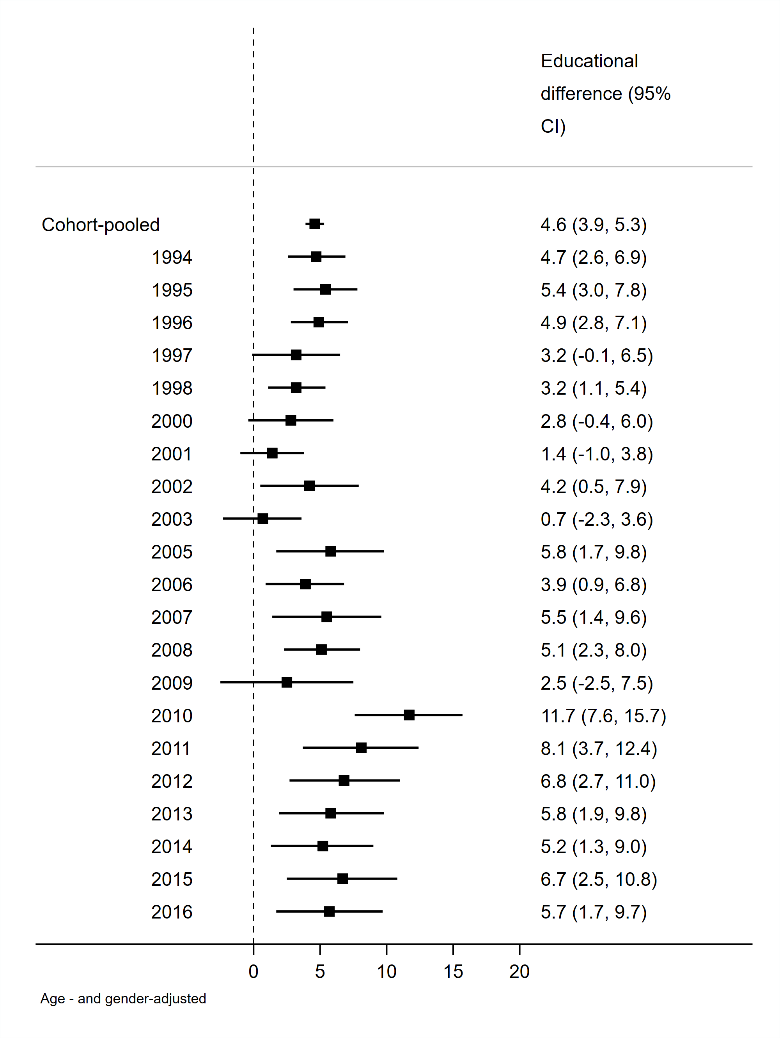


Fig. S3**. Education-related differences in blood pressure lowering medication use (%) in midlife (42-46 years, from birth cohort data, left panel) and across adulthood (≥25 years, from repeated cross-sectional data, right panel).** Note: estimates are the Slope Index of Inequality (absolute difference in outcome between the lowest and highest educational attainment groups). An SII of zero (vertical line) indicates equity in antihypertensive medication use. Cohort studies: estimates adjusted for child and adult social class indicate potential cumulative associations over the life course.


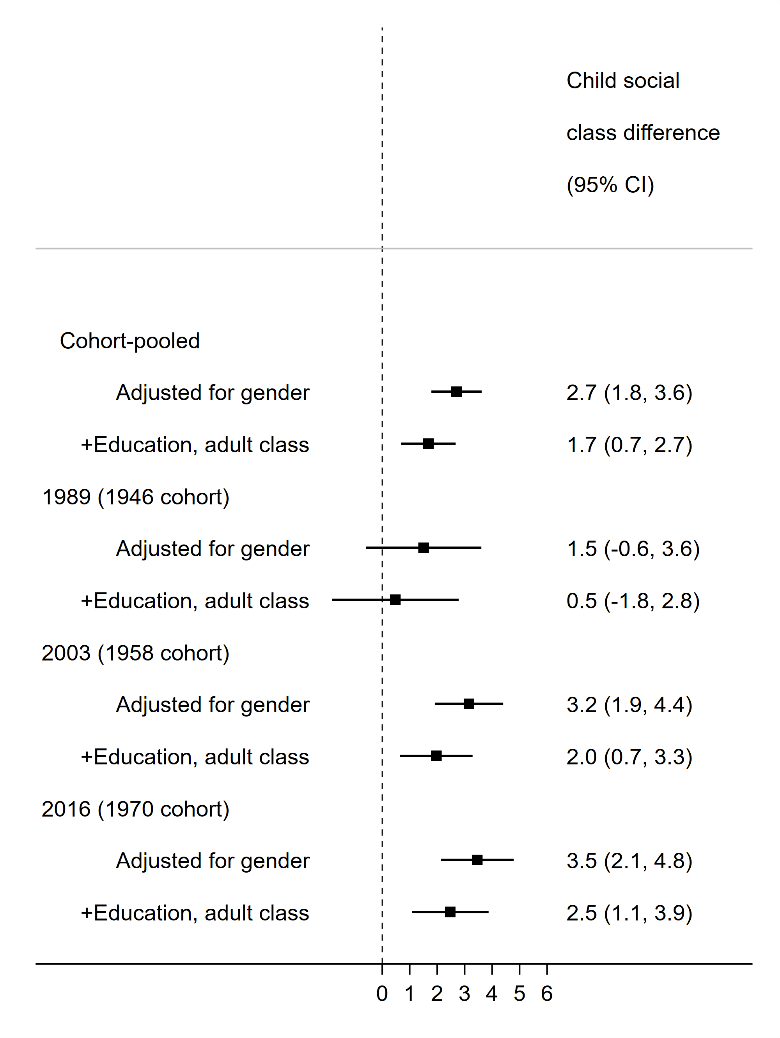

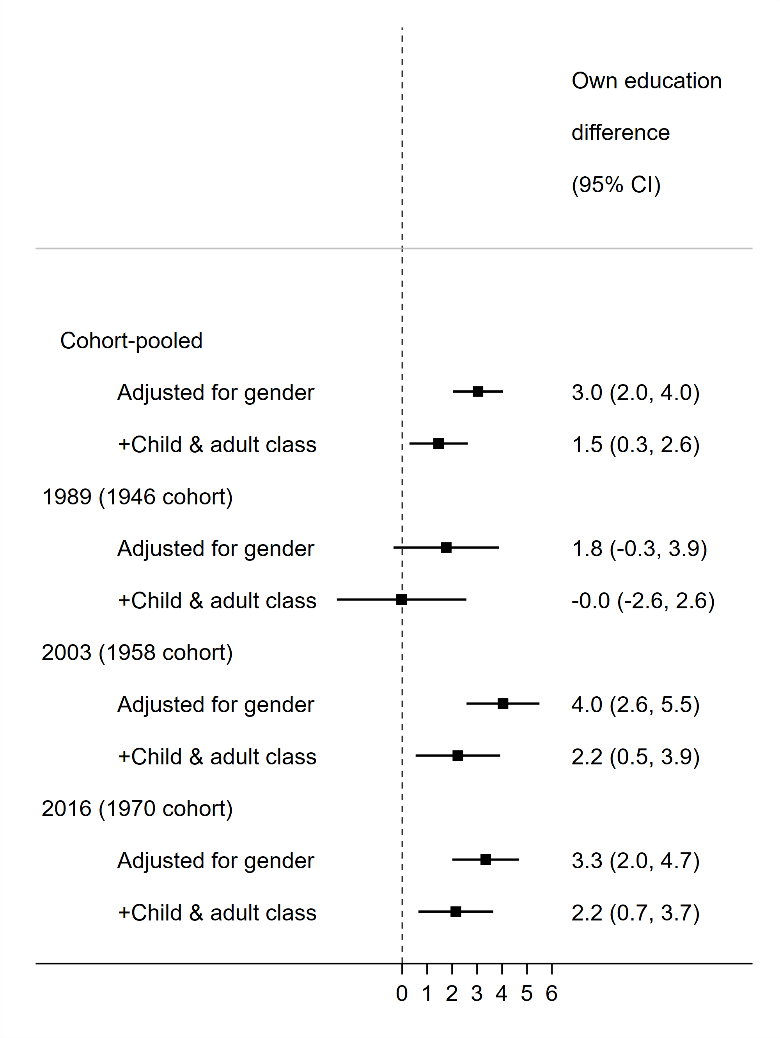

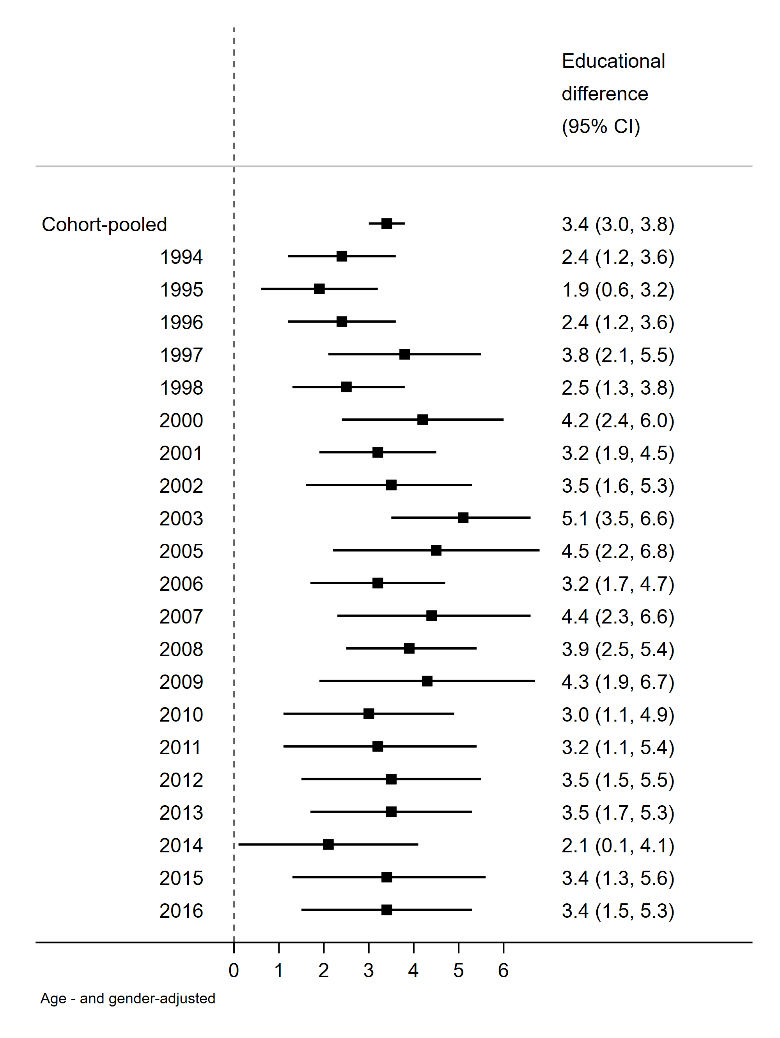


Fig. S4**. Socioeconomic position across life and mean difference in systolic blood pressure (mmHg) in midlife (42-46 years, from birth cohort data, left panels) and across adulthood (≥25 years, from repeated cross-sectional data, right panel); without adjusting for blood pressure-lowering treatment (i.e. observed rather than underlying BP).** Note: estimates are the Slope Index of Inequality (absolute difference in mean SBP levels between the lowest and highest educational attainment groups). An SII of zero (vertical line) indicates equity in SBP levels. Cohort analyses: estimates adjusted for own education and adult social class indicate potential cumulative associations over the life course. The comparable estimates which did adjust for blood-pressure lowering treatment using the constant addition method are shown in Figure 1 and 2.


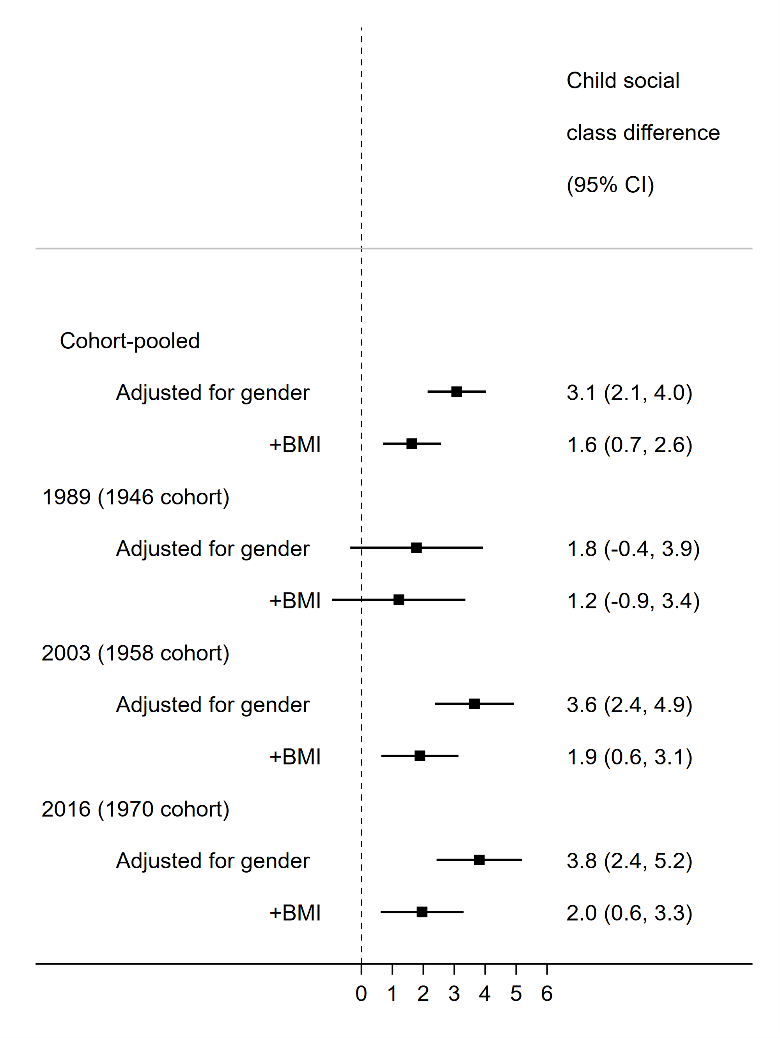

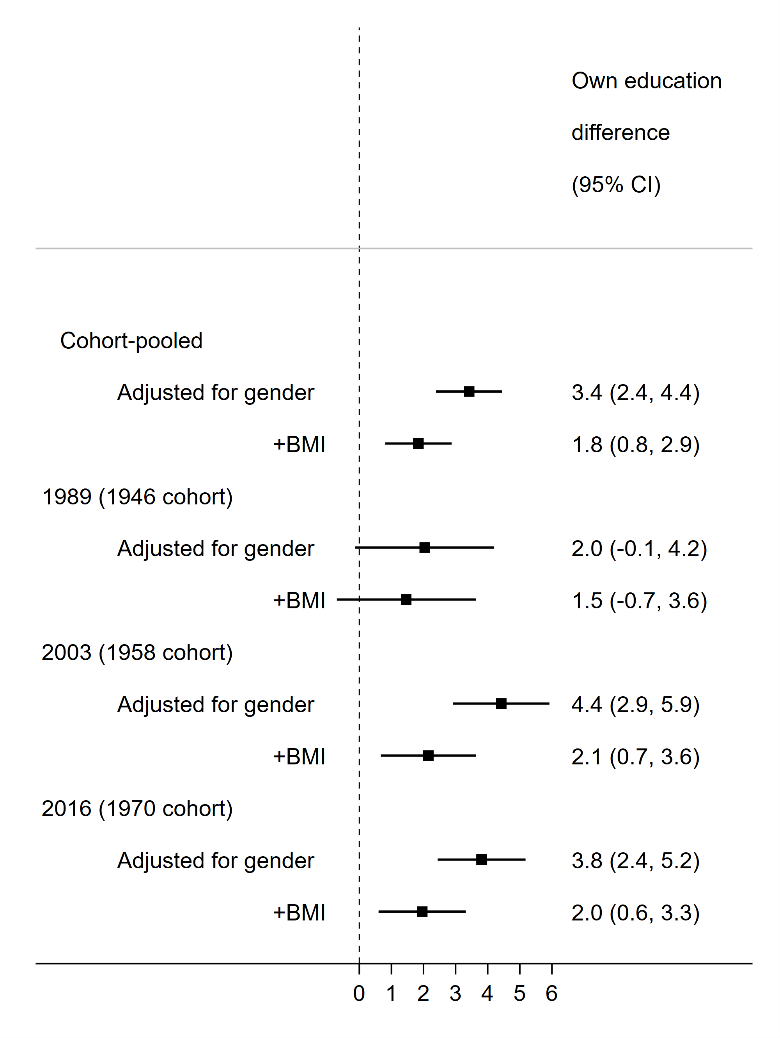

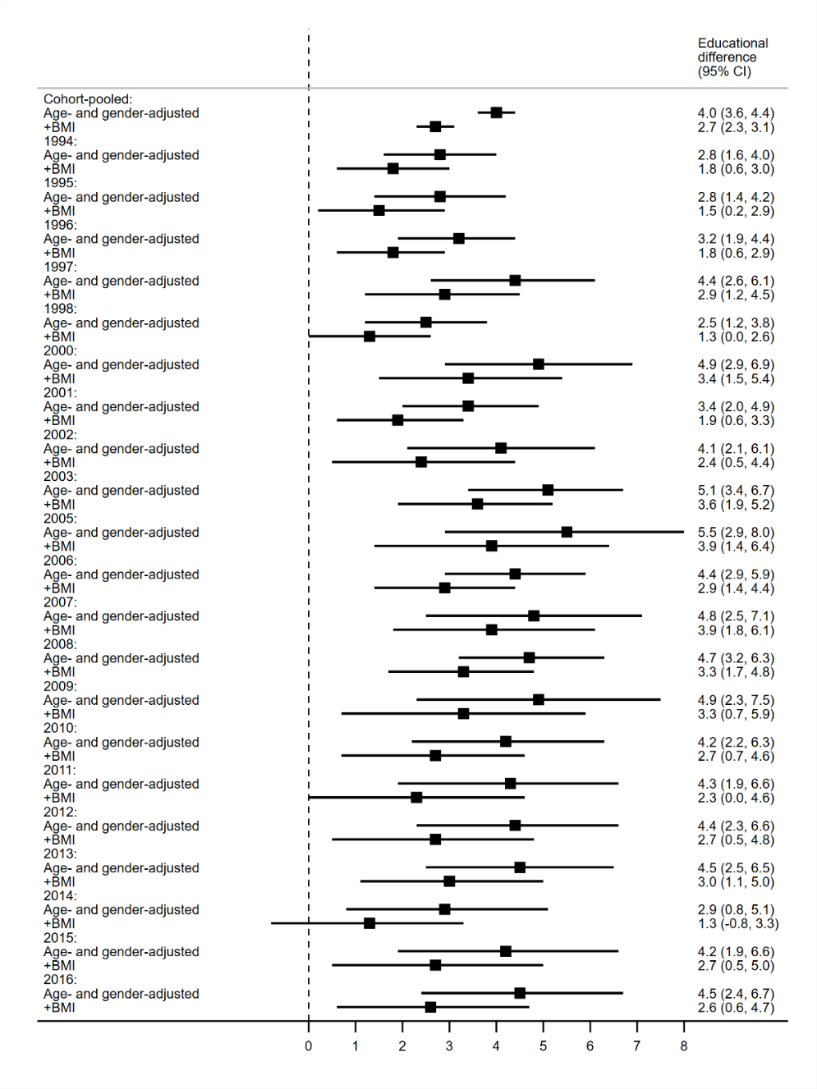


Fig. S5**. Socioeconomic position and mean difference in systolic blood pressure (mmHg) in midlife (42-46 years, from birth cohort data, left panel) and across adulthood (≥25 years, from repeated cross-sectional data, far right panel)—before and after adjustment for body mass index (BMI).** Note: estimates are the Slope Index of Inequality (absolute difference in mean SBP levels between the lowest and highest socioeconomic position). An SII of zero (vertical line) indicates equity in BP levels. Underlying SBP levels obtained by adding a constant of 10mmHg to those using antihypertensive medication. Cohort analyses: estimates adjusted for child and adult social class indicate potential cumulative associations over the life course.

**Females (cohorts) Males (cohorts) Females (HSE) Males (HSE)**


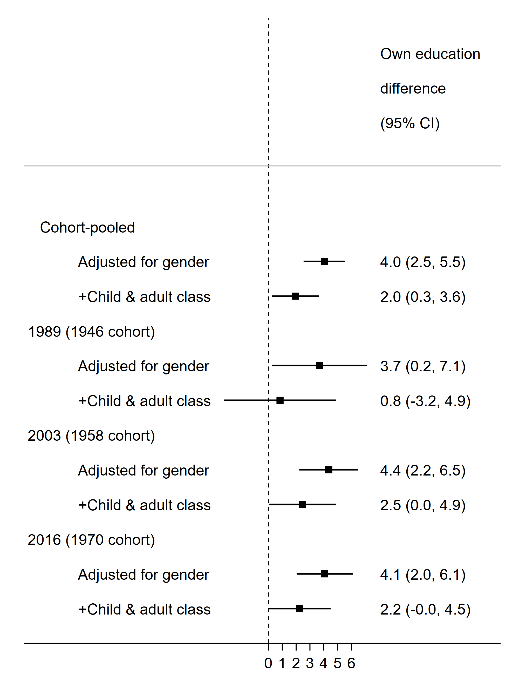

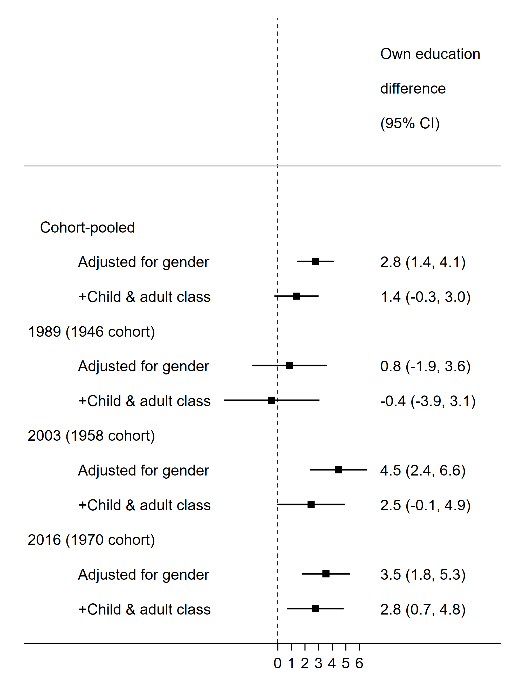

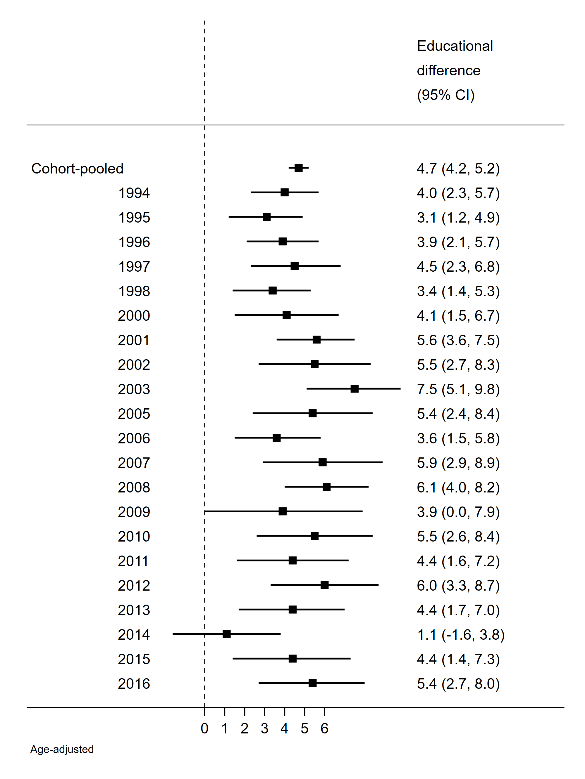

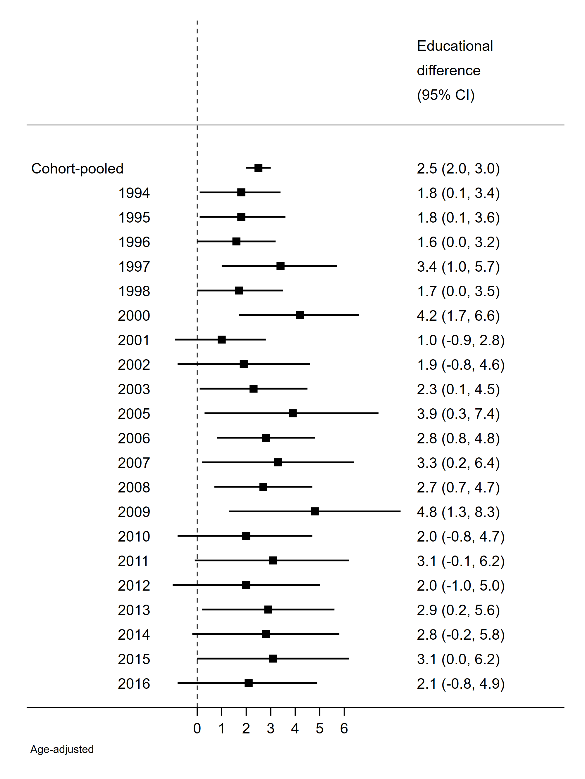


Fig. S6**. Early life socioeconomic position and mean difference in systolic blood pressure (mmHg) in midlife (42-46 years, from birth cohort data, left panels) and across adulthood (≥25 years, from repeated cross-sectional data, right panel); analyses stratified by gender.** Note: estimates are the Slope Index of Inequality (absolute difference in outcome between the lowest and highest educational attainment groups).

**Females (cohorts) Males (cohorts)**


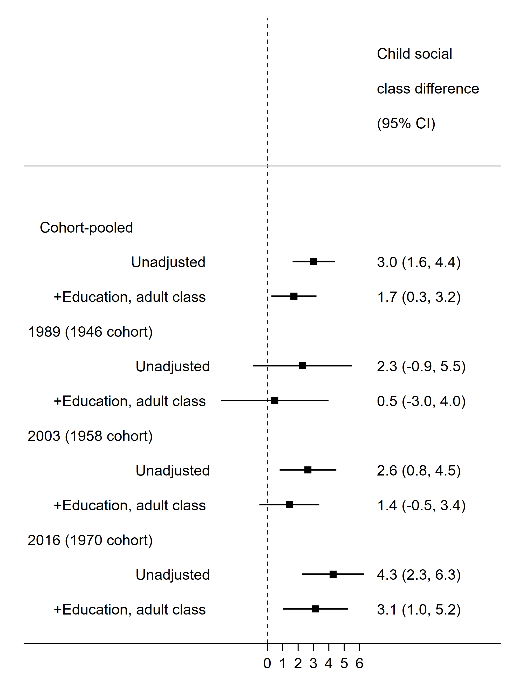

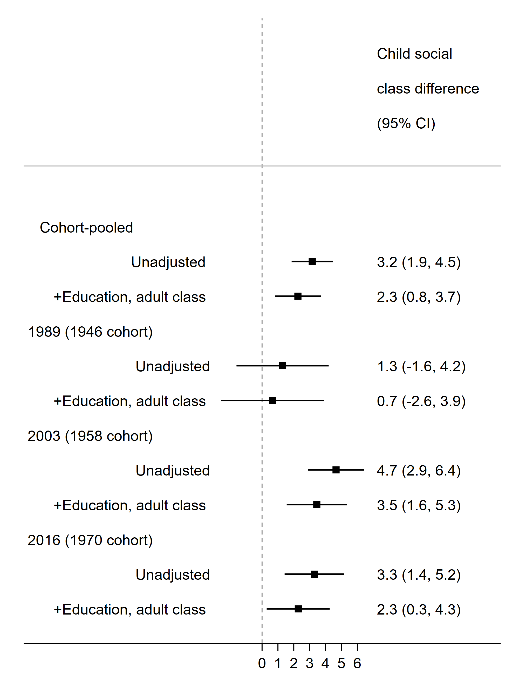


**…Fig. S6 continued.**


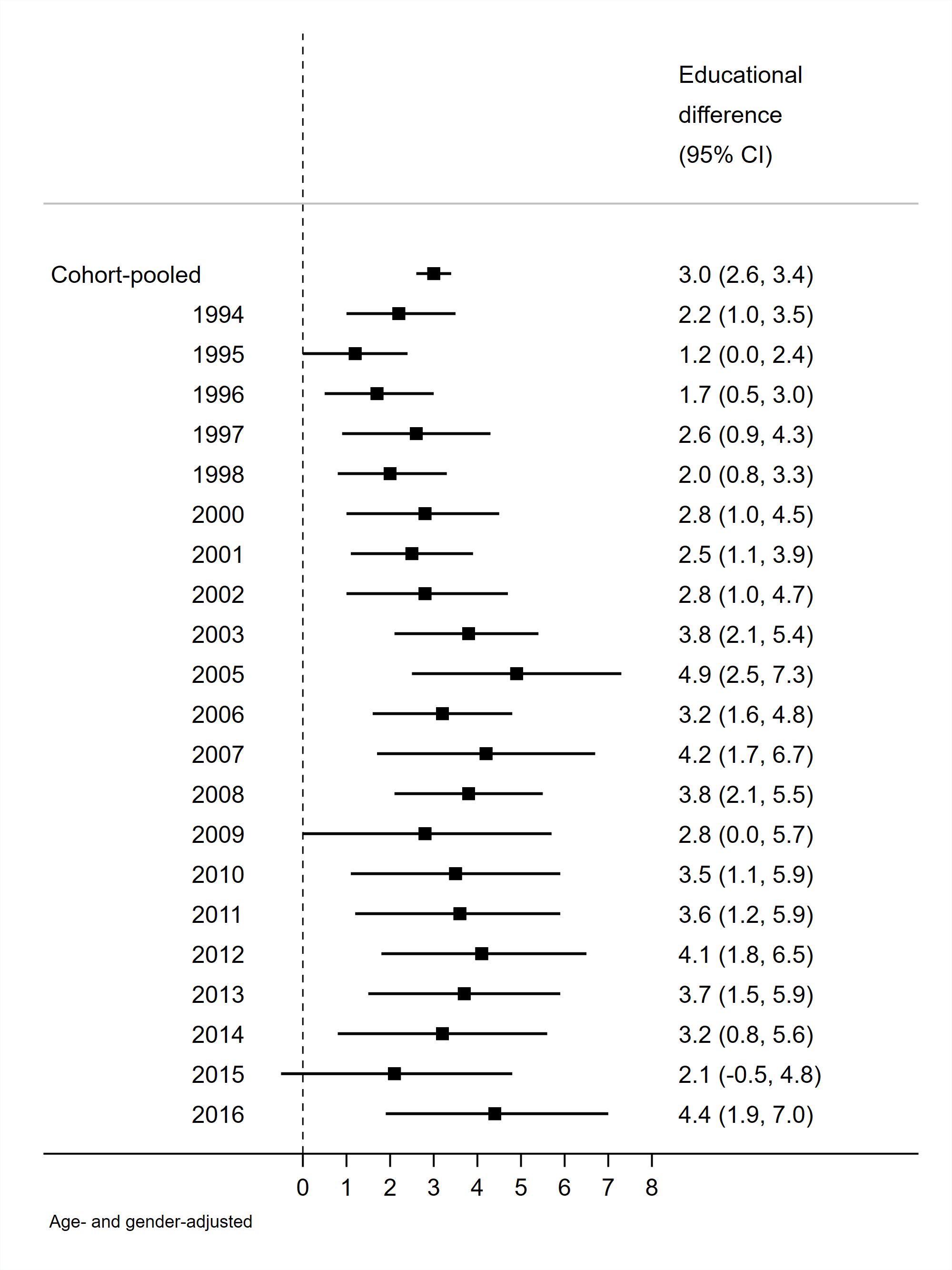

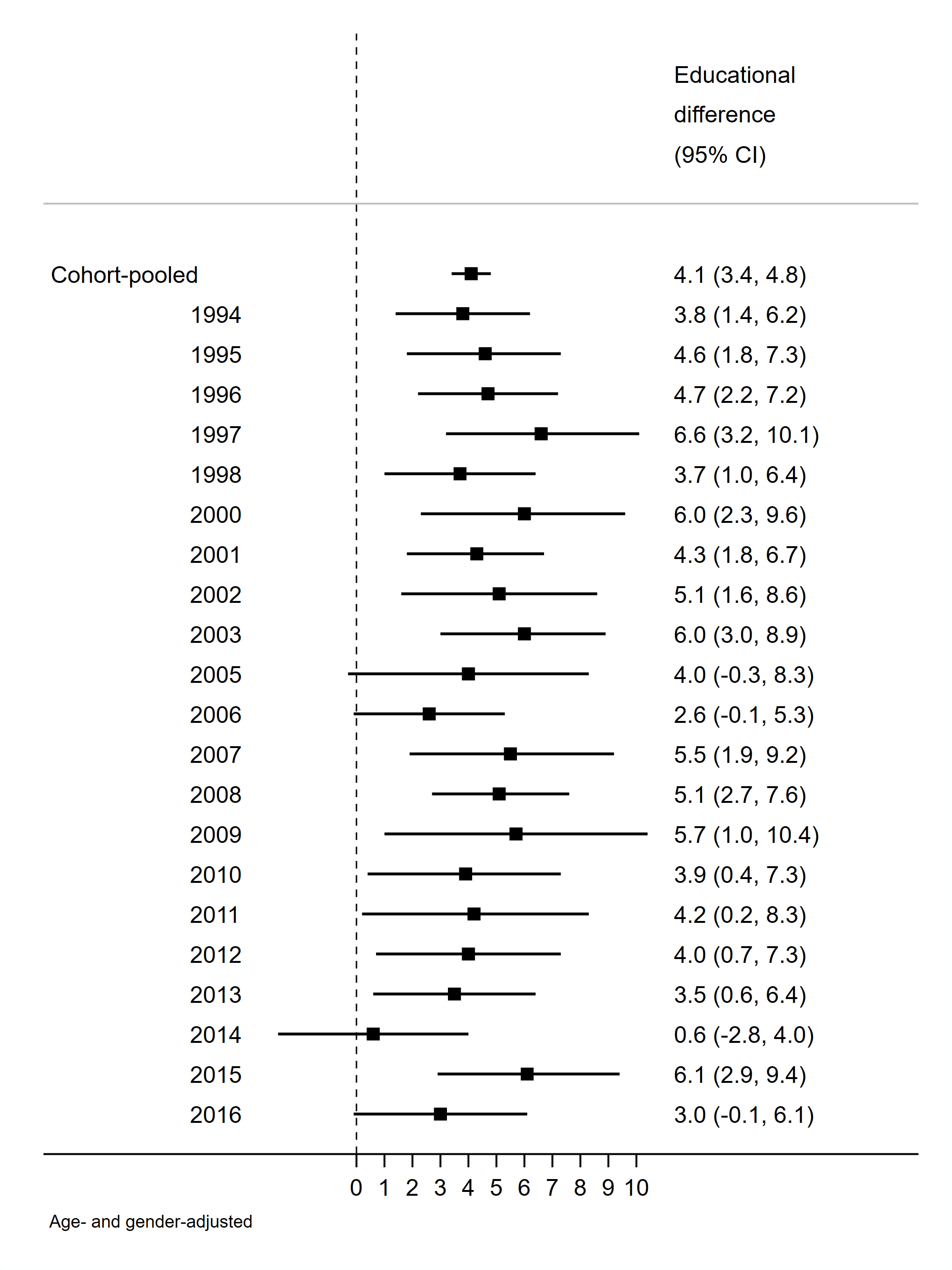


Fig. S7**. Education-related difference in mean systolic blood pressure (mmHg) in those aged 25-54 years (left panel) or 55 years and older (right panel); data from repeated cross-sectional data.** Note: estimates are the Slope Index of Inequality (absolute difference in outcome between the lowest and highest educational attainment groups).
